# Merged gastrointestinal stromal tumor, intestinal obstruction, and perforation in Meckel’s diverticulum: Case report

**DOI:** 10.1097/MD.0000000000046257

**Published:** 2026-05-12

**Authors:** YuXi Gao, Fang Hu, Yan Xia, Liang Lv

**Affiliations:** a Department of General Surgery, The Affiliated Hospital of Qingdao University, Qingdao, China; b Department of Stomatology, The Affiliated Hospital of Qingdao University, Qingdao, China; c Department of Pathology, The Affiliated Hospital of Qingdao University, Qingdao, China.

**Keywords:** case report, complication, gastrointestinal stromal tumors, Meckel’s diverticulum

## Abstract

**Rationale:**

Gastrointestinal stromal tumor (GIST) arising in Meckel’s diverticulum represents a rare condition; tumor enlargement may result in intestinal obstruction and perforation. Previous case reports have documented atypical imaging features associated with this pathology.

**Patient concerns:**

We report a case involving a 67-year-old male patient with intestinal obstruction and perforation secondary to a GIST located within a Meckel’s diverticulum.

**Diagnoses:**

The patient was diagnosed with malignant GISTs within Meckel’s diverticulum, complicated by perforation and intestinal obstruction.

**Interventions:**

Computed tomography imaging clearly displays the imaging features of Meckel’s diverticulum tumor and its complications.

**Outcomes and lessons:**

Computed tomography imaging clearly delineated a cystic protrusion corresponding to Meckel’s diverticulum, identified the tumor boundary within the diverticulum, and visualized the supplying vascular structures as well as the imaging features associated with complications such as obstruction and perforation. This detailed diagnostic approach facilitates prompt and precise diagnosis, thereby enabling the development of timely and targeted treatment strategies.

## 1. Introduction

Meckel’s diverticulum is a relatively common congenital anomaly of the digestive tract, with an incidence of approximately 2%. Although most cases remain asymptomatic and are incidentally discovered during surgical procedures, clinical manifestations may arise when complications – such as gastrointestinal bleeding, inflammation, or intestinal obstruction – occur, thereby complicating preoperative diagnosis.^[1]^ This report presents a case of Meckel’s diverticulum complicated by tumor formation, perforation, and intestinal obstruction. Notably, several imaging features in this case were more pronounced than those typically observed, including the cystic protrusion of the diverticulum, well-defined tumor boundaries within the diverticulum, distinct visualization of blood supply vessels, and characteristic imaging findings associated with complications such as obstruction and perforation. The imaging observations of cystic protrusion of Meckel’s diverticulum, tumor boundary in the diverticulum, and visualization of blood supply vessel structure emphasize the importance of rapid diagnosis and effective treatment planning for this complex symptom.

## 2. Case report

**Figure 1.**
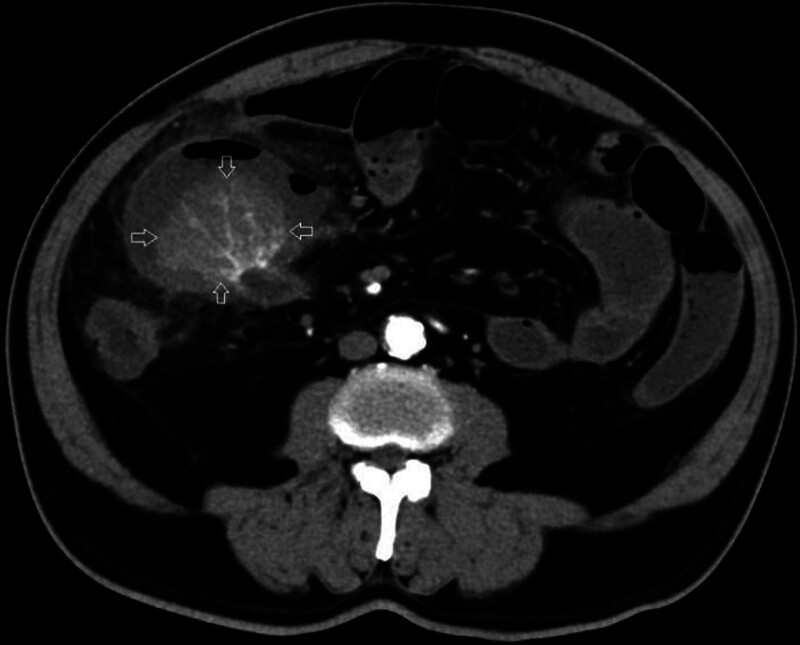
A cystic lesion measuring approximately 60 mm by 44 mm is observed along the antimesenteric border of the ileum in the lower right abdominal quadrant.

**Figure 2.**
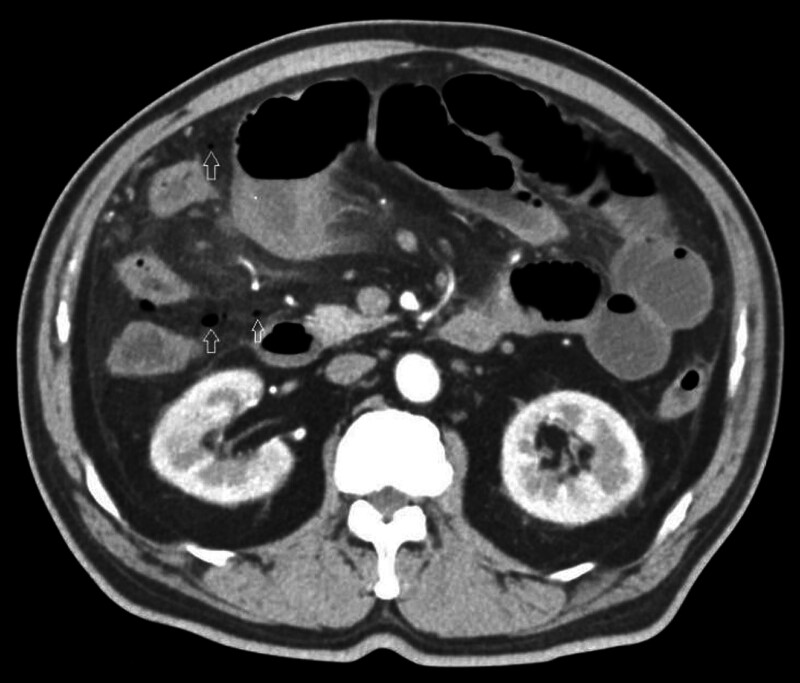
Observations reveal the presence of both free fluid and gas densities within the abdominal cavity.

A 67-year-old male patient presented with abdominal distension and progressively worsening pain that began 3 days prior, with no identifiable inciting factor. On examination, localized tenderness was noted in the midabdomen, while other assessments were unremarkable. The patient was managed conservatively without initial treatment. Contrast-enhanced CT of the abdomen revealed a pouch-like protrusion on the anterior wall of the terminal ileum in the right lower quadrant, adjacent to a soft tissue mass measuring 60 mm × 44 mm. Multiple vascular structures were observed within the mass (Fig. 1, axial view, indicated by straight arrows). Additionally, signs of proximal small bowel obstruction and free intraperitoneal gas were noted (Fig. 2, indicated by straight arrows). Contrast-enhanced CT scan shows suspected Meckel’s diverticulum with tumor (neuroendocrine tumor or gastrointestinal stromal tumor?) combined with diverticulum perforation and small intestinal obstruction. Based on these clinical and radiologic findings, a diagnosis of small intestinal diverticular tumor with perforation was considered. The tumor is suspected to be either a neuroendocrine or mesenchymal neoplasm; however, definitive diagnosis requires further pathological confirmation.

**Figure 3.**
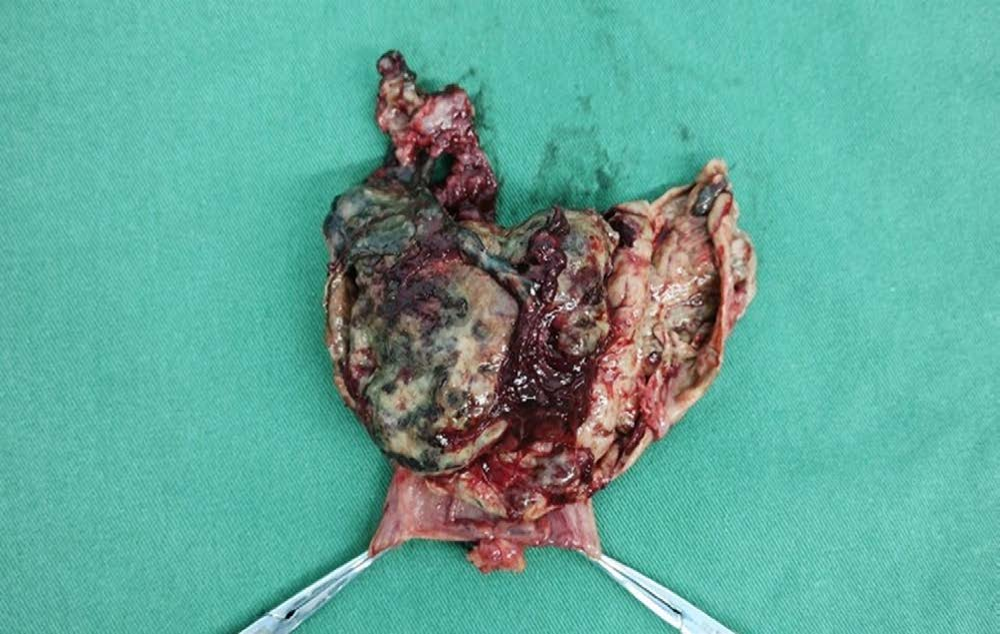
The tumor was located in the ileum, 1.5 m from the cecum, and measured 6 × 6 × 4 cm. It resulted in a rupture of the diverticulum.

**Figure 4.**
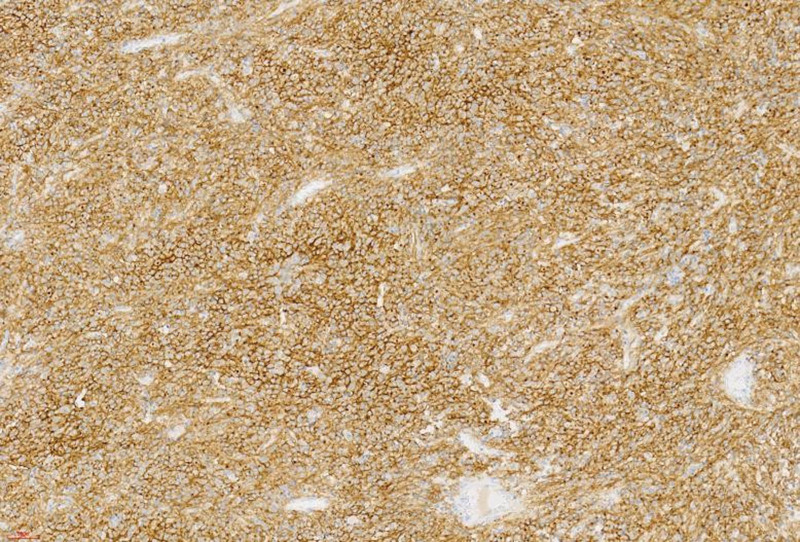
The tumor is located within the submucosal to serosal layers of the ileum and exhibits areas of hemorrhage and necrosis. Immunohistochemical staining reveals positive expression of CD117.

The patient underwent surgical resection of an ileal tumor, during which a 6 × 6 × 4 cm mass was identified in the ileum approximately 1.5 m from the cecum (see Fig. 3). The mass’s capsule had spontaneously ruptured. Postoperatively, the tumor underwent pathological examination, immunohistochemical analysis, and genetic testing. Histologically, the tumor was located from the submucosal to the serosal layers of the ileum, with focal areas of hemorrhage and necrosis; mitotic figures were observed at a frequency of approximately 1 per 5 mm^2^. Immunohistochemical staining yielded positive results for CD117 and DOG-1, while CD34, SMA, Desmin, ALK (5A4), CD30, S100, and STAT6 were negative (see Fig. 4). A c-kit exon mutation (K550-K558del) was detected, and the tumor was classified as high risk. The patient was diagnosed with malignant gastrointestinal stromal tumor (GIST) within Meckel’s diverticulum, complicated by perforation and intestinal obstruction. Postoperatively, the patient resumed oral intake on the fourth day and was discharged on the seventh day. Currently, the patient is receiving oral imatinib mesylate, reports no discomfort, maintains a normal diet and sleep pattern, and exhibits good physical strength. A follow-up abdominal enhanced computed tomography (CT) scan obtained 3 months after surgery revealed no evidence of tumor recurrence or metastasis. Given the high risk of recurrence, the patient is scheduled for follow-up over 3 years, with evaluations every 3 months, including abdominal enhanced CT and comprehensive blood tests.

## 3. Discussion

The asymptomatic Meckel’s diverticulum lacks specific features on plain x-ray, barium examinations, CT scans, and other imaging modalities. A presumptive diagnosis is typically only possible when complications arise.^[2]^ In cases where Meckel’s diverticulum is complicated by intestinal obstruction, perforation, or mass effects, imaging may reveal findings such as intestinal dilatation, intra-abdominal free gas, and space-occupying lesions on plain films, barium studies, or CT scans. Doppler ultrasound has limited diagnostic utility for Meckel’s diverticulum, while ultrasonography may detect a fluid-filled, tubular diverticulum located away from the cecum along with signs of invagination, localized bowel wall thickening, diverticular wall swelling, or a pelvic abscess.^[3]^ In recent years, advanced techniques such as capsule endoscopy, double-balloon enteroscopy, magnetic resonance enterography, and CT enterography have significantly improved the diagnosis of small bowel diseases, including Meckel’s diverticulum. Although diagnostic laparoscopy or laparotomy remains an effective and safe method for lesion localization and surgical removal of Meckel’s diverticulum, these procedures are invasive and are generally reserved for cases where other diagnostic evidence supports their use.^[4]^

Meckel’s diverticulum is typically asymptomatic and often identified incidentally during imaging studies or abdominal surgical procedures. However, when complications such as hemorrhage, obstruction, or perforation arise, its clinical presentation may closely resemble that of other intraperitoneal pathologies – including alternative etiologies of small bowel obstruction or inflammatory bowel disease – thereby complicating the differential diagnosis.^[5]^

In previous case reports on Meckel’s diverticulum tumors, CT scans primarily revealed abdominal pneumoperitoneum or intestinal obstruction, with the final diagnosis confirmed during intraoperative exploration.^[6]^ In contrast, enhanced abdominal CT in this case clearly delineated the cystic protrusion of Meckel’s diverticulum, the tumor boundary within the diverticulum, and the associated vascular supply, enabling an accurate preoperative diagnosis. Small bowel obstruction is the most common complication of Meckel’s diverticulum in adults, with inflammatory complications – namely, diverticulitis and perforation – occurring in approximately 20% of cases.^[7]^ Although tumors within Meckel’s diverticulum are rare and predominantly benign, malignant cases, including carcinoid tumors, mesenchymal tumors, and adenocarcinomas, have been reported.^[8]^ GIST account for 20% to 30% of tumors in the small intestine. In the present case, perforation and intestinal obstruction were attributed to a mesenchymal tumor arising within the diverticulum.

The management of complicated Meckel’s diverticulum is primarily surgical. Typically, a wedge resection of the diverticulum is performed, often followed by an end-to-end anastomosis of the ileal segment. In instances where a malignant tumor is present, an extensive resection of the small intestine along with its mesenteric tissues may be warranted.^[9]^ With recent advancements in endoscopic methods, double-balloon enteroscopy has emerged as a viable option for treating Meckel’s diverticulum bleeding or achieving full-thickness resection in cases of inverted Meckel’s diverticulum.^[10]^ The necessity of surgical intervention for incidentally detected, asymptomatic Meckel’s diverticulum remains a subject of debate. Some surgeons now utilize risk scoring systems to inform the decision-making process. Notably, Robijn et al proposed a decision scoring system based on 4 risk factors – patient age under 45 years, male gender, the presence of a fibrous band, and a diverticulum length exceeding 2 cm – recommending resection when the total score is 6 or higher.^[11]^

Tumor rupture, whether occurring spontaneously or during surgery, is associated with an almost 100% risk of recurrence. Therefore, patients experiencing tumor rupture should be regarded as having metastatic disease and treated appropriately with imatinib. However, in particular cases, such as tumor micro-perforation, the duration of imatinib treatment may warrant separate consideration.^[12]^

## 4. Conclusion

This medical record is diagnosed as malignant GIST within Meckel’s diverticulum, complicated by perforation and intestinal obstruction. Meckel’s diverticulum is the most prevalent gastrointestinal developmental anomaly, although complications are infrequent, its clinical presentation can be challenging as it is difficult to distinguish from other acute abdominal conditions based solely on symptoms. CT imaging clearly displays the imaging features of Meckel’s diverticulum tumor and its complications. CT imaging is a diagnostic tool that can quickly obtain and diagnose diseases for patients with acute abdomen. This detailed diagnostic approach facilitates prompt and precise diagnosis, thereby enabling the development of timely and targeted treatment strategies.

## Acknowledgments

The authors thank the patient for agreement to the publication of the report.

## Author contributions

**Data curation**: Yan Xia.

**Formal analysis**: Fang Hu.

**Investigation**: Yu Xi Gao, Yan Xia.

**Methodology**: Fang Hu.

**Resources**: Yan Xia.

**Visualization**: Yan Xia.

**Writing – review & editing**: Yu Xi Gao, Liang Lv.
